# Test–Retest Reproducibility of Reduced-Field-of-View Density-Weighted CRT MRSI at 3T

**DOI:** 10.3390/tomography10040038

**Published:** 2024-03-29

**Authors:** Nicholas Farley, Antonia Susnjar, Mark Chiew, Uzay E. Emir

**Affiliations:** 1 School of Health Sciences, Purdue University, West Lafayette, IN 47907, USA; farleyn@purdue.edu; 2 Weldon School of Biomedical Engineering, Purdue University, West Lafayette, IN 47907, USA; asusnjar@mgh.harvard.edu; 3 Department of Medical Biophysics, University of Toronto, Toronto, ON M5G 1L7, Canada; mark.chiew@utoronto.ca

**Keywords:** non-Cartesian, nuclear, magnetic, resonance, spectroscopy, imaging, test–retest, reproducibility

## Abstract

Quantifying an imaging modality’s ability to reproduce results is important for establishing its utility. In magnetic resonance spectroscopic imaging (MRSI), new acquisition protocols are regularly introduced which improve upon their precursors with respect to signal-to-noise ratio (SNR), total acquisition duration, and nominal voxel resolution. This study has quantified the within-subject and between-subject reproducibility of one such new protocol (reduced-field-of-view density-weighted concentric ring trajectory (rFOV-DW-CRT) MRSI) by calculating the coefficient of variance of data acquired from a test–retest experiment. The posterior cingulate cortex (PCC) and the right superior corona radiata (SCR) were selected as the regions of interest (ROIs) for grey matter (GM) and white matter (WM), respectively. CVs for between-subject and within-subject were consistently around or below 15% for Glx, tCho, and Myo-Ins, and below 5% for tNAA and tCr.

## 1. Introduction

Non-invasive measurements of neurometabolites such as N-acetyl aspartate (NAA), a marker of neuronal loss/dysfunction; creatine (Cr), a marker for cell membrane turnover; glutamate (Glu), the primary excitatory neurotransmitter; and y-aminobutyric acid (GABA), the primary inhibitory neurotransmitter, are important for studying and diagnosing cranial diseases which lack an anatomical explanation. Proton magnetic resonance spectroscopy (1H-MRS) is one of the physical means to non-invasively measure neurometabolic concentrations in vivo, making them essential for investigating neurological, neuropsychological, and oncological conditions [1]. Previous MRS studies have revealed significant differences in metabolic levels for cases of acute stroke, chronic multiple sclerosis, and brain tumors compared with a healthy brain [2]. Additional metabolites arise in specific conditions, such as succinate and acetate in abscesses, lipids in various abnormalities, propylene glycol after administration of parenteral preparations, or ethanol after alcohol consumption [2]. Since its conception, single-voxel-MRS (SV-MRS) has remained the preferred modality for quantifying neurometabolic concentrations, mostly for its fast acquisition and simple execution. However, the spectra are susceptible to partial-volume effects when different tissues (i.e., grey matter (GM), white matter (WM), etc.) are within the measurement volume. Alternatively, multi-voxel-MRS (MRSI) can measure spectra from different regions at the same time, and with better resolution than SV-MRS, but requires longer acquisitions to compensate for its inherently lower signal-to-noise ratio (SNR).

Conventionally, MRSI acquisitions were Cartesian because of their widespread use in standard magnetic resonance imaging (MRI). Recently, many non-Cartesian trajectories have been proposed and demonstrated to sample the spatial and spectral dimensions efficiently [3]. For example, the self-rewinding nature of concentric ring trajectories (CRTs) reduces the total acquisition duration by avoiding deadtimes associated with other trajectories [4,5]. It can be augmented to improve spectral bandwidth (SBW) by reconstructing interleaved trajectories produced via repeated measurements with different phase offsets. Theoretically, equidistant CRTs (e-CRTs) can fully sample k-space twice as fast as echo-planar spectroscopic imaging (EPSI), since an arbitrary number of rings (N) of e-CRTs can be substituted for a corresponding 2N rows of EPSI [1], but at the cost of poorly sampling the outer regions of k-space. More recently, it has been proposed to vary the distance between sampling radii according to a density-weighting (DW) formula, which may be tuned to improve outer k-space coverage in such a manner as to mimic a standard Hanning filter [6]. Nominal resolutions of 110 μL and 36 μL have been reported for 3D-MRSI using DW-CRTs at 3T, with an improvement to 20 μL achieved in 17 min at 7T [7], and for 2D-MRSI, a similar nominal resolution of 24 μL was obtained in 46 min at 9.4T [8]. Additionally, CRT spatial–spectral Encoding (SSE) has been observed to resist the appearance of aliasing (or “wrap-around”) artifacts for situations where it would normally be expected, such as for EPSI when the field of view (FOV) is less than the size of the patient’s head. In more recent work, a reduced FOV-^1^H-MRSI (rFOV or ZOOM MRSI) sequence was proposed, which uses a combination of semi-adiabatic localization by adiabatic selective refocusing (semi-LASER), metabolite cycling, and DW-CRTs to measure localized metabolite signals without the appearance of aliasing artifacts [9]. Since the rFOV requires fewer encoding steps (number of rings) to obtain higher resolutions, a higher nominal resolution and a faster acquisition time were achieved. The reported 2.5 × 2.5 × 10 mm^3^ (62.5 μL) resolution spectroscopic images were reconstructed using data acquired within 9.5 min at 3T [9]. To evaluate the reproducibility of this method, this study aims to determine the within-subject and between-subject reproducibility of the rFOV-DW-CRT-MRSI technique, as measured through a voxel-by-voxel coefficient of variance (CV) analysis on data acquired in the posterior cingulate cortex (PCC) and right superior corona radiata (SCR) accomplished using a multi-session test–retest protocol.

## 2. Materials and Methods

### 2.1. Volunteers and Hardware

Data were acquired using a Siemens Prisma 3T MR system (Siemens, Erlangen, Germany) with a 64-channel head array receive coil and four volunteer subjects who did not present any neurological, neurodegenerative, or psychiatric disorders. Approval from Purdue’s Institutional Review Board and written/informed consent from all subjects were obtained before the MR examinations. The four volunteers (2 M|2 F) were recruited from our institution’s (Purdue University, West Lafayette, IN, USA) student population. All were between the ages of 18 and 22, and the reported medical conditions during the pre-experimentation debriefing were not relevant.

### 2.2. Data Acquisition

T_1_-weighted MPRAGE images were acquired for each subject with the following parameters: field of view (FOV) = 240 × 240 × 176 mm^3^, TR = 1900 ms, TE = 2.13 ms, TI = 900 ms, flip angle = 8, 176 transverse slices, 0.9 × 0.9 × 1 mm^3^ voxels. The rFOV-MRSI scan was planned on the T_1_-weighted images to acquire data from the PCC and SCR using the parameters described in Emir et al. [9], with rFOV = (120 mm × 120 mm × 10 mm), in-plane matrix of 48 × 48 for a nominal voxel resolution of (2.5 mm × 2.5 mm × 10 mm), semi-LASER localization, TR = 1500 ms, and TE = 32 ms. DW-CRTs were used for 2D-MRSI data sampling, with 64 points per ring, 24 rings, 4 interleaves, and a final spectral bandwidth (SBW) of 1.25 kHz with 256 spectral points [9]. Instead of traditional water-suppression, metabolite cycling was used by applying either a standard or inverse excitation profile for non-water resonances. When executed correctly, a water-suppressed spectrum can be calculated by subtracting the corresponding spectra [10]. Metabolite cycling reduces scan time by removing long pulse trains of water-suppression pulses [11]. The volume of interest (VOI) was centered on the posterior cingulate cortex. No angle was applied to the VOI versus the transverse plane. First- and second-order B_0_-shimming was performed through two iterations of Siemens’ manual convergence method (GRESHIM) to optimize the coefficients for shimming coils in the VOI. A linewidth of less than 30 Hz was obtained with this shimming protocol for all four subjects. After completion, the subject was removed from the scanner for 15 min before being sent back inside for a second set of identical scans.

### 2.3. Data Processing

Spectroscopic k-space data were sampled via a non-Cartesian DW-CRT SSE [6,10]. Raw files were exported offline for pre-processing, reconstruction, and quantification. The adjoint NUFFT (non-uniform fast Fourier transform) was used for reconstruction [6,12,13]. The NUFFT operator used the k-space frequencies known a priori by mathematically simulating the k-space trajectories with a sampling rate of 64 points per ring. Data were therefore reconstructed along the 2D spatial plane into a 48 × 48 grid of uniform Cartesian spacing. Data were pre-processed according to Emir et al. [9]. A Gaussian filter was applied in the time domain for all spectra using a coefficient of 250 ms. The non-water-suppressed “upfield” and “downfield” metabolite cycled spectra were used for frequency and phase corrections for each voxel. These two spectra from every voxel were then subtracted to recover the metabolic signal and added to recover a strong water reference signal. Metabolite quantification was performed using the LCModel package (version 6.3-1M) as described in Emir et al. [9]. The basis spectra used for fitting included 8 LCModel-simulated macromolecule resonances (at 0.91, 1.31, 1.43, 1.67, 1.95, 2.08, 2.25, and 3 ppm), as described in Steel et al. [14]. Additionally, the basis spectra included model spectra of alanine, aspartate, ascorbate/vitamin C, glycerophosphocholine, phosphocholine (PCho), creatine (Cr), phosphocreatine (PCr), GABA, glucose, glutamine, (Gln), glutamate (Glu), glutathione, lactate, and myo-inositol (myo-ins). Spectral fitting was performed between 0.5 and 4.2 ppm. Once completed, 5 major neurometabolic groups were selected for further analysis due to having Cramer–Rao lower bounds (CRLB) less than 15% for voxels within the relevant ROIs. These were the total choline (tCho), total creatine (tCr), combined glutamate and glutamine (Glx), myo-inositol (Myo-Ins), and combined NAA and NAA-glutamate (tNAA). Of these, the quantification results from each voxel were used to create metabolite maps for the water-reference concentration and tCr ratio. These metabolic maps were then co-registered with their corresponding T1-weighted MPRAGE image. 

**Figure 1:**
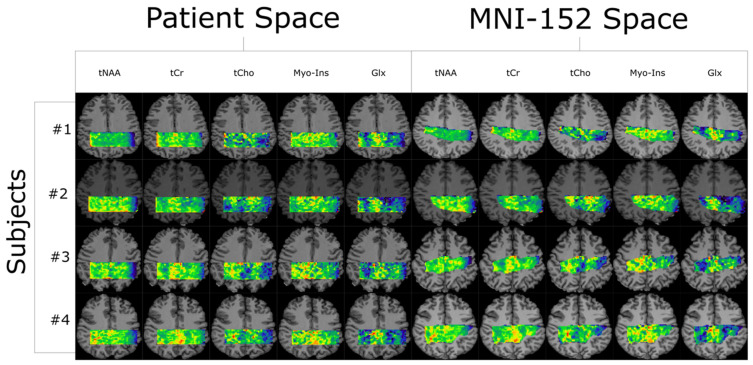
Quantification results from LCModel are plotted as five metabolite maps for each of the four subjects shown in both their native-patient space and transformed into MNI-152. All color maps are relative intensity with respect to each subject and metabolite. The # symbol refers to the word “number”.

**Figure 2:**
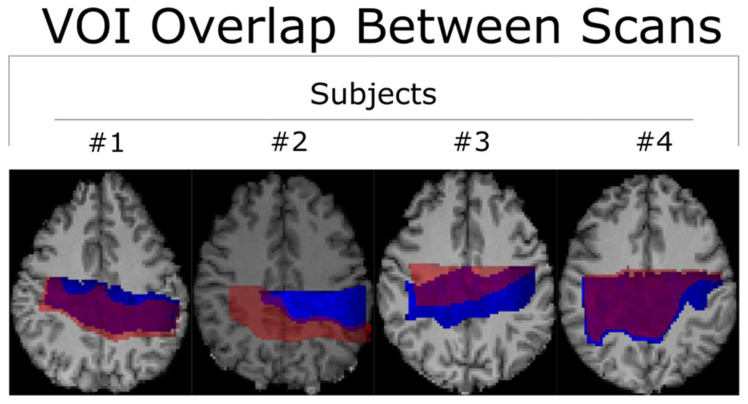
Two scans for each subject were acquired and then converted into MNI-152 space to facilitate direct comparison between voxels. The first and second scans are represented in blue and semi-transparent red, respectively. The # symbol refers to the word “number”.

**Figure 3:**
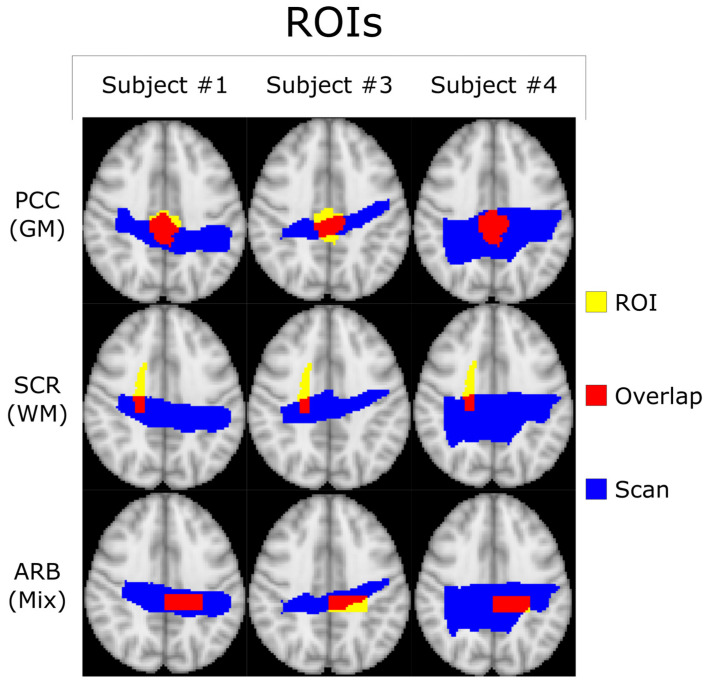
Transverse profiles of the standard MNI-152-2mm-Brain with the PCC and SCR ROIs defined by the Harvard–Oxford Cortical Atlas (GM) and the JHU ICBM-DTI-81 White-Matter Labels Atlas (WM). Voxels where the ROI and the subject’s scan overlap are highlighted in red and included within the statistical analysis. There is also a third arbitrary (ARB) ROI selected to demonstrate the generalizability of the technique. The # symbol refers to the word “number”.

All co-registered metabolite maps and T_1_-weighted MPRAGE images were converted into MNI-152 to facilitate voxel alignment between scans of different subjects. A direct comparison between MNI-152 and patient space metabolite maps can be seen in Figure 1. A shared coverage map for each subject was created by using one scan’s tNAA metabolite map to mask that of the other. Masking refers to filtering out voxels correlated with values less than or equal to zero in another corresponding (masking) 2D image. Each subject’s MNI-152 spatial overlap between their first and second spectroscopic imaging scans is shown in Figure 2. Subject 2′s overlap was noticeably smaller than that of other subjects, which is why different masks were used for both scans to increase the number of voxels used in the statistical measurements. In contrast, the overlap of Subject 4 was noticeably greater than the others. A graphic depicting the overlap between a subject’s cross-coverage map and the target ROI (GM and WM) is shown in Figure 3. The PCC was chosen as the basis for an ROI in GM. Its MNI-152 representation was made by taking every voxel from region 30 of the Harvard–Oxford Cortical Atlas and digitally masking them with the subject’s shared-coverage mask. Likewise, the SCR was chosen as the basis for an ROI in WM, and its MNI-152 representation was made by taking every voxel from region 25 of the JHU ICBM-DTI-81 White-Matter Labels atlas and digitally masking them with the subject’s shared-coverage mask.

In addition to the water-reference and tCr-ratio MNI-152 metabolite maps previously mentioned, a separate set of tissue-weighted metabolic maps was generated by correcting the water-reference values for the tissue deviations in each voxel using the formula recommended in the LCModel manual [15,16]:

$$w_{conc}=\frac{43,300f_{GM}+35,880f_{WM}+55,556f_{CSF}}{1-f_{CSF}}$$

Here, ***f*** is the partial volume fraction of a specific tissue type, as denoted by its subscript. The partial volume fractions for each voxel were estimated using FSL’s segmentation function (FAST) [17] to decompose each subject’s MNI-152 T_1_-weighted MPRAGE image into tissue-fraction maps (number of input categories = 1, number of classes = 3). With these parameters, the three maps correlate to GM, WM, and cerebrospinal fluid (CSF). 

Furthermore, another separate set of metabolite maps was produced by normalizing the water-reference metabolite maps in each voxel using the total metabolic contribution:

$$n_{i}=\frac{s_{i}}{\sqrt{\sum_{1}^{5}{\left(s_{i}\right)}^{2}}}$$

Here, ***n*** is the desired normalized metabolic concentration, ***s*** is the water-reference metabolic concentration, and the subscript ***i*** denotes one of the five major neurometabolites of interest (1:tCho, 2:tCr, 3:Glx, 4:Ins, 5:tNAA).

### 2.4. Statistical Analysis of Reproducibility

Both within-subject variance and between-subject variance for every combination of ROI, metabolite, and quantification ratio were measured by the corresponding CV. This study defined CV as the ratio between the sample’s standard deviation and the mean. A sample consisted of the subjects’ first and second scans when measuring their specific within-subject CV. Metabolic concentration values for all voxels within an ROI were averaged together to calculate the ROI-specific metabolic concentration. The mean, standard deviation, and CV were determined from ROI-averaged metabolic concentrations for every subject. The within-subject CVs from all subjects were then averaged to calculate the group’s within-subject coefficient of variance for both ROIs. For between-subject variance, it proceeded similarly, but with a sample combining the first scan from each subject.

## 3. Results

**Figure 4:**
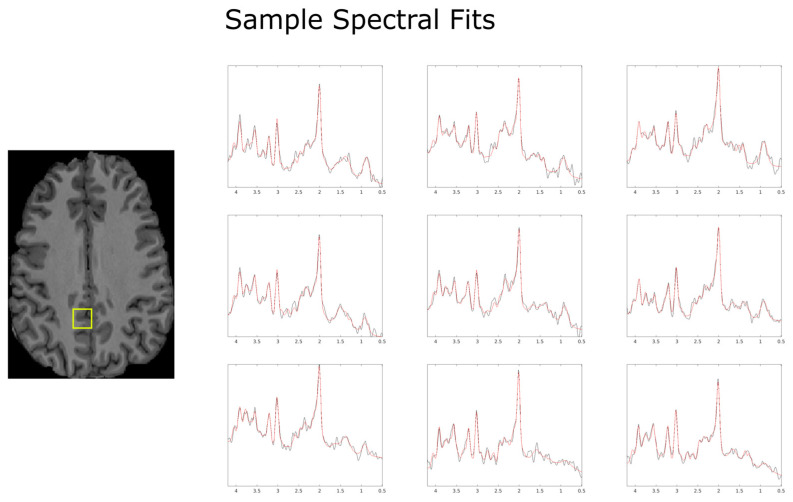
Example spectral reconstructions (red) produced by LCModel using the specifics detailed in the methods sections, overlayed with the pre-processed spectrum (black). Data come from the first scan of Subject 1 and are presented as a 3 × 3 sample window for 9 spectra roughly centered in the localization volume. The locations of the spectra presented above are found within the yellow box overlayed in the anatomical image above.

All subjects completed the acquisition protocol. Since the total amount of overlapping voxels for Subject 2 was significantly lower than for Subjects 1, 3, and 4, as may be seen in Figure 2, different regions of interest were used for each of the two scans to increase the sample size and improve the statistical analysis. Examples of LCModel spectral fits overlayed with the measured spectra for subject 1 are displayed within Figure 4.

### Within-Subject and Between-Subject Reproducibility

The within-subject CVs for both ROIs are given in Table 1. LCModel determined the CRLB to be <5% for tNAA and tCr and <20% for tCho, Glx, and Myo-Ins for every voxel within the two ROIs for all four subjects. The CVs for tCho and Myo-Ins were consistently higher in GM than in WM. By contrast, Glx was noticeably higher in WM as opposed to GM. Furthermore, the within-subject CVs for tCr and tNAA were relatively identical for both ROIs. The between-subject CVs for all ROIs, metabolites, and quantification methods can also be found in Table 1. On average, the between-subject CV was greater than its corresponding within-subject CV. The relative differences between the average within-subject CVs and between-subject CVs were greater for metabolites contributing a larger portion of the total signal. 

## 4. Discussion

This study performed a multi-session MRSI reproducibility experiment for rFOV-MRSI by acquiring two scans from four volunteers at 3T and then analyzing the statistical variance between and within subjects. The mean, standard deviation, and CV were calculated for each subject after using masks to select voxels corresponding only to the PCC or the SCR, which served as ROIs for GM and WM, respectively. These regions were selected for their predominantly homogeneous composition of their corresponding tissue type within the center and their heterogenous periphery. This study exploited the relatively high nominal resolution of the voxels to create irregular volumes that conformed with the target ROI’s shape and size. Emulating these irregular shapes for SVS-MRS and MRSI with >3 mm resolution would be difficult, if not implausible, due to the structural variations occurring at <3 mm scales.

Concentration maps with nominal voxel resolutions of 62.5 μL (2.5 × 2.5 × 10 mm^3^) were produced using 2D-MRSI data within a clinically feasible acquisition time of 9.5 min on a Siemens 3T Prisma scanner. rFOV-DW-CRTs for sampling made this possible with their fast acquisition rate, intrinsically high-spectral resolution, and resistance to aliasing artifacts [9]. Despite the relatively small voxel volume and shorter acquisition duration, this study reports the within-subject CV for tNAA and tCr to be lower than what has been reported elsewhere for SV-MRS reproducibility studies at 1.5T and 3T [18,19], and is comparable to MRSI reproducibility studies at 7T [13], with 3.1% and 2.9% for tNAA and tCr, respectively, and also at 9.4T [20], with 5.6% for NAA/tCr.

The rFOV used in this study’s MRSI sequence contributed to a more homogenous magnetic field within every voxel by reducing the spatial volume that the shimming algorithm needed to optimize. Furthermore, reducing the nominal voxel size reduced the spectral linewidth, which offset some of the sensitivity losses caused by the decreased signal-to-noise ratio from smaller voxels [21]. Even further, the removal of voxels through a digital mask improved the magnetic field homogeneity and intra-voxel linewidth, which was only possible to reproduce across all subjects through MNI-152 space. Many previous test-reproducibility studies have relied on anatomical markers as the primary method to reproduce voxel placement across different scans, subjects, scanners, and medical centers [19,20,22,23,24,25,26,27]. While still used here, this study additionally transformed all scanner data (anatomical and spectroscopic) into MNI-152 to facilitate alignment between voxels from different scans and subjects [26,27]. 

**Figure 5:**
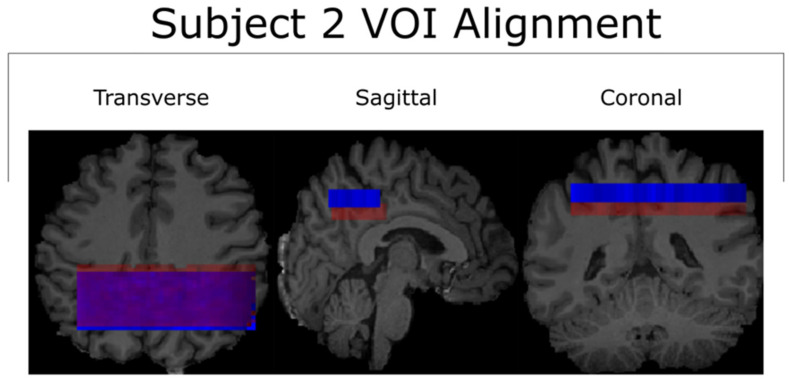
All three viewing angles for the VOI alignment of both scans for Subject 2. The misalignment between the two is visible along the sagittal and coronal planes. This misalignment was the greatest contributor to the small cross-coverage in the corresponding MNI-152 space, as seen in Figure 2. The test and retest scans for subject 2 are represented by the colors red and blue respectively.

The relatively small spatial overlap between the first and second scans of Subject 2 after being transformed into MNI-152 space is likely due to the poor placement of the second scan’s VOI in native-patient space. The sagittal viewing plane in Figure 5 shows that the location of the second scan (blue) was anatomically superior to that of the first scan (red), despite the nearly complete overlap along the transverse plane. The cause of this discrepancy was operator error at the time of the VOI’s placement. Incidentally, this supports the notion that a non-operator-based method of aligning voxels (such as MNI-152) is necessary for quality control in reproducibility studies [28].

The calculated within-subject CVs suggest that the variance was below the maximum threshold permitted to resolve actual variations in metabolic concentrations across scans. For example, the inverse relationship between the CVs for Glx and tCho (when comparing WM and GM) [14,19,20,22,23,24,25] can be seen from the data displayed in Table 1. Furthermore, Glx was found to have a higher variance in WM versus GM (16.1% vs. 10.0%) as opposed to tCho and Myo-Ins, where the opposite was seen to hold (8.5% vs. 11.0% and 8.8% vs. 12.1%, respectively). A higher level of variance is expected from metabolites with a lower total signal contribution, which is what other literature has reported for both grey and white matter [22,23,25]. Disease-related alterations to metabolite levels can be detected with 95% confidence if these alterations are greater than twice the Cramer–Rao lower bound [15]. Within-subject and between-subject CVs for all five major neurometabolites investigated were comparable with their average CRLBs calculated using LCModel. This suggests that low variations in metabolic concentrations were detectable. Since relevant pathology is known to cause alterations in metabolic concentrations, the alterations characteristic of different pathologies should also be detectable.

Calculated between-subject CVs are, on average, greater than their within-subject counterparts for all combinations of ROIs and metabolites. This is expected from any form of measurement that demonstrates a significant level of specificity, and suggests the rFOV-DW-CRT’s acquisition/reconstruction protocol can reliably differentiate between separate subjects.

The difference between the average within-subject CVs and the average between-subject CVs was relatively larger for metabolites that contributed a greater fraction of the total signal (e.g., tCr (2.5% vs. 6.0% (GM) and 3.8% vs. 6.2% (WM)), and tNAA (2.0% vs. 5.5% (GM) and 2.9% vs. 5.3% (WM))).

In addition to the variability associated with the protocol itself, other potential sources of variability could include subject motion, frequency drift from a change in temperature, and scanner instabilities. The subject motion was minimized by reducing the amount of time they needed to be within the machine to no more than 20 min at a time by taking them out halfway through, as described in the methods section. Furthermore, the gap between the test and retest scans permitted the MRI machine to partially cool off and thus minimize the corresponding frequency drift normally observed at higher temperatures during the retest scan.

It is worth noting that, while the sample size for this study was relatively small (*n* = 4), it is not uncommon to have such sample sizes in MRS(I) test–retest studies for magnetic field strengths between 3T and 9.4T [1,20,23,26,29,30,31,32]. However, a discussion on its potential impact regarding error estimates for the CVs reported in Table 1 is warranted. The estimated error for each CV value is, on average, about 40% of the calculated value, as is characteristic of a sample size of *n* = 4. Therefore, this study’s reported CVs across different metabolites and tissue types can conceivably be attributed to statistical variation. However, the repeated patterns of metabolite and tissue type observed in the data (i.e., lower CV_tCho_ in white matter versus grey matter) across multiple methods of quantification (i.e., absolute, normalized, tCr ratio, and tissue-factor correction) provide evidence supporting the notion that the observed trends in CV values across tissue types are reflective of changes in actual signal intensity as opposed to a random statistical error intrinsic to the estimation.

This study’s acquisition protocol is constrained to only two dimensions, while 3D MRSI protocols have been proposed and documented previously in the literature [1]. If desired, the sequence can easily be converted into 3D through the addition of another phase-encoding dimension or through the concept of simultaneous multi-slice (SMS)-MRSI [33]. However, 2D-MRSI sequences, by restricting the size of the VOI, have an easier time shimming than their 3D counterparts since they only analyze a necessarily smaller segment of the brain, thereby allowing for our high resolution and relatively fast acquisition times.

The rFOV-DW-CRT MRSI protocol investigated in this study may have potential for clinical applications in the foreseeable future. However, there are aspects which can be improved through additional research, namely, an investigation into the viability of reducing the number of CRTs during analysis to thereby further decrease the total amount of time necessary to sample a full set of k-space. Progress in this direction should increase the protocol’s clinical utility, which would then increase the likelihood of its integration within the greater hospital workflow.

## 5. Conclusions

A combination of rFOV-DW-CRT SSE, metabolite cycling for water suppression, and semi-laser localization were simultaneously used to acquire in-plane nominal voxel resolutions of 2.5 × 2.5 mm^2^ at 3T within a clinically feasible scan time of 9.5 min. Our study has quantified this protocol’s within-subject and between-subject reproducibilities through their CVs and found them to be similar to what is reported for other MRSI modalities.

## Figures and Tables

**Table 1:** Calculated within-subject and between-subject CVs for every combination of ROI, metabolite, and method used to report the data. Within-subject CVs were, on average, lower than their between-subject counterparts, with the greatest discrepancies occurring for tCr and tNAA.

Within-Subject CV (%)			Metabolites		
Versus Method					
**Grey Matter**	tCho	tCr	Glx	Ins	tNAA
Absolute	11.0 ± 4.7	1.8 ± 0.76	10.0 ± 4.3	12.1 ± 5.2	2.0 ± 0.84
Normalized	10.4 ± 4.4	4.0 ± 1.7	9.7 ± 4.1	14.7 ± 6.3	0.9 ± 0.37
tCr Ratio	10.6 ± 4.5	-	11.7 ± 5.0	12.1 ± 5.2	2.7 ± 1.1
TF Correction	11.7 ± 5.0	1.7 ± 0.72	9.3 ± 3.9	13.4 ± 5.7	2.2 ± 0.93
**White Matter**	tCho	tCr	Glx	Ins	tNAA
Absolute	8.5 ± 3.6	4.2 ± 1.8	16.1 ± 6.9	8.8 ± 3.7	1.9 ± 0.80
Normalized	8.8 ± 3.7	3.2 ± 1.4	11.9 ± 5.1	13.3 ± 5.7	3.9 ± 1.6
tCr Ratio	9.7 ± 4.1	-	13.0 ± 5.6	14.1 ± 6.0	4.0 ± 1.7
TF Correction	8.8 ± 3.7	4.1 ± 1.7	16.1 ± 6.9	8.8 ± 3.7	1.9 ± 0.80
**Mixed Composition**	tCho	tCr	Glx	Ins	tNAA
Absolute	10.0 ± 4.2	3.4 ± 1.4	12.3 ± 5.2	3.8 ± 1.6	3.2 ± 1.4
Normalized	11.0 ± 4.7	3.2 ± 1.4	18.1 ± 7.8	4.4 ± 1.9	5.0 ± 2.1
tCr Ratio	8.6 ± 3.6	-	11.9 ± 5.1	7.2 ± 3.0	2.7 ± 1.1
TF Correction	10.1 ± 4.3	3.2 ± 1.4	12.0 ± 5.1	7.8 ± 3.3	3.0 ± 1.3
**Between-Subject CV (%)**			**Metabolites**		
**Versus Method**					
**Grey Matter**	tCho	tCr	Glx	Ins	tNAA
Absolute	12.0 ± 5.1	7.0 ± 3.0	13.4 ± 5.7	12.8 ± 5.5	6.7 ± 2.8
Normalized	10.7 ± 4.6	3.7 ± 1.6	8.3 ± 3.5	13.4 ± 5.7	3.7 ± 1.6
tCr Ratio	11.3 ± 4.8	-	10.2 ± 4.3	12.4 ± 5.3	4.0 ± 1.7
TF Correction	12.5 ± 5.3	7.2 ± 3.0	12.5 ± 5.3	14.4 ± 6.2	7.6 ± 3.2
**White Matter**	tCho	tCr	Glx	Ins	tNAA
Absolute	13.1 ± 5.6	8.2 ± 3.5	18.0 ± 7.8	11.4 ± 4.9	4.9 ± 2.1
Normalized	10.7 ± 4.6	3.4 ± 1.4	15.3 ± 6.6	12.6 ± 5.4	5.2 ± 2.2
tCr Ratio	10.2 ± 4.3	-	16.9 ± 7.3	13.1 ± 5.6	6.3 ± 2.7
TF Correction	12.4 ± 5.3	7.0 ± 3.0	17.5 ± 7.5	11.2 ± 4.8	4.9 ± 2.1
**Mixed Composition**	tCho	tCr	Glx	Ins	tNAA
Absolute	12.8 ± 5.5	5.9 ± 2.5	17.1 ± 7.4	6.3 ± 2.7	5.2 ± 2.2
Normalized	14.4 ± 6.2	6.0 ± 2.5	23.2 ± 10.2	8.6 ± 3.6	7.7 ± 3.3
tCr Ratio	10.6 ± 4.5	-	17.8 ± 7.7	13.0 ± 5.6	5.1 ± 2.2
TF Correction	11.9 ± 5.1	5.8 ± 2.5	21.4 ± 9.3	14.4 ± 6.2	5.0 ± 2.1

## Data Availability

All data, tables, and figures in this manuscript are original, and data are available upon request from the corresponding authors, as they have not been uploaded to an online database.
